# Effects of the Total Saponins from *Rosa laevigata* Michx Fruit against Acetaminophen-Induced Liver Damage in Mice via Induction of Autophagy and Suppression of Inflammation and Apoptosis

**DOI:** 10.3390/molecules19067189

**Published:** 2014-05-30

**Authors:** Deshi Dong, Lina Xu, Xu Han, Yan Qi, Youwei Xu, Lianhong Yin, Kexin Liu, Jinyong Peng

**Affiliations:** 1 College of Pharmacy, Dalian Medical University, Western 9 Lvshunnan Road, Lvshunkou District, Dalian 116044, Liaoning Province, China; 2 The First Affiliated Hospital of Dalian Medical University, Dalian 116011, China; 3 Research Institute of Integrated Traditional and Western Medicine of Dalian Medical University, Dalian 116011, China

**Keywords:** acetaminophen, hepatoprotective effect, liver injury, *Rosalaevigata* Michx, total saponins

## Abstract

The effect of the total saponins from *Rosa* *laevigata* Michx fruit (RLTS) against acetaminophen (APAP)-induced liver damage in mice was evaluated in the present paper. The results showed that RLTS markedly improved the levels of liver SOD, CAT, GSH, GSH-Px, MDA, NO and iNOS, and the activities of serum ALT and AST caused by APAP. Further research confirmed that RLTS prevented fragmentation of DNA and mitochondrial ultrastructural alterations based on TdT-mediated dUTP nick end labeling (TUNEL) and transmission electron microscopy (TEM) assays. In addition, RLTS decreased the gene or protein expressions of cytochrome P450 (CYP2E1), pro-inflammatory mediators (IL-1β, IL-4, IL-6, TNF-α, iNOS, Bax, HMGB-1 and COX-2), pro-inflammatory transcription factors (NF-κB and AP-1), pro-apoptotic proteins (cytochrome C, p53, caspase-3, caspase-9, p-JNK, p-p38 and p-ERK), and increased the protein expressions of Bcl-2 and Bcl-xL. Moreover, the gene expression of IL-10, and the proteins including LC3, Beclin-1 and Atg5 induced by APAP were even more augmented by the extract. These results demonstrate that RLTS has hepatoprotective effects through antioxidative action, induction of autophagy, and suppression of inflammation and apoptosis, and could be developed as a potential candidate to treat APAP-induced liver damage in the future.

## 1. Introduction

Acetaminophen (APAP) is a widely used analgesic and antipyretic drug, and overdoses of it can cause hepatic necrosis and renal failure [1]. This drug is metabolized by cytochrome P450 to produce *N*-acetyl-*p*-benzoquinoneimine (NAPQI), which can react with glutathione (GSH) to cause oxidative stress that may trigger the mitochondrial signal pathway and lead to cell injury [2,3,4,5]. Mitochondrial damage is a well-known feature caused by APAP, which can inhibit mitochondrial respiration and decrease membrane potential [6,7] to produce mitochondrial dysfunction and oxidant stress [8,9]. In addition, some biological processes including apoptosis, inflammatory and autophagy have been found in APAP-induced hepatotoxicity [10,11,12,13,14].

APAP-induced hepatotoxicity can cause some serious diseases [15]. *N*-acetylcysteine (NAC) is the only used drug to treat the liver damage caused by APAP [16], and however, NAC is limited to treat APAP-induced liver injury because it has to be given at the early stage [17]. Therefore, exploration of new potential candidates to treat APAP-induced liver damage is urgent. Nowadays, natural products with high efficiency and low toxicity have attracted more and more attentions, and some natural components including silymarin, liquiritigenin and arjunolic acid have been found to be beneficial for treatment of APAP-induced liver injury [18,19,20,21].

*Rosa laevigata* Michx, a famous medicinal plant, is widely distributed in China. The fruit of this plant has been widely used for improving kidney health, inhibiting arteriosclerosis and reducing inflammation [22,23]. Saponins are considered to be the major active constituents [24] with anti-oxidant, anti-fungal and anti-viral actions [25]. Our previous study has indicated that the total saponins from this herb showed significant hepatoprotective effects against carbon tetrachloride-induced acute liver injury in mice [26]. However, the effect of the total saponins from *R. laevigata* Michx fruit (RLTS) against APAP-induced liver injury has not been reported in the best of our knowledge.

The aim of the present study was to evaluate the action of RLTS against liver injury in male Kunming mice caused by APAP and then the possible mechanisms were also tested. We found that RLTS showed potential protective effect against APAP-induced liver damage through induction of autophagy, suppression of inflammation and apoptosis.

## 2. Results and Discussion

### 2.1. Effects of RLTS on Serum ALT, AST Activities and Liver Histopathology

**Figure 1:**
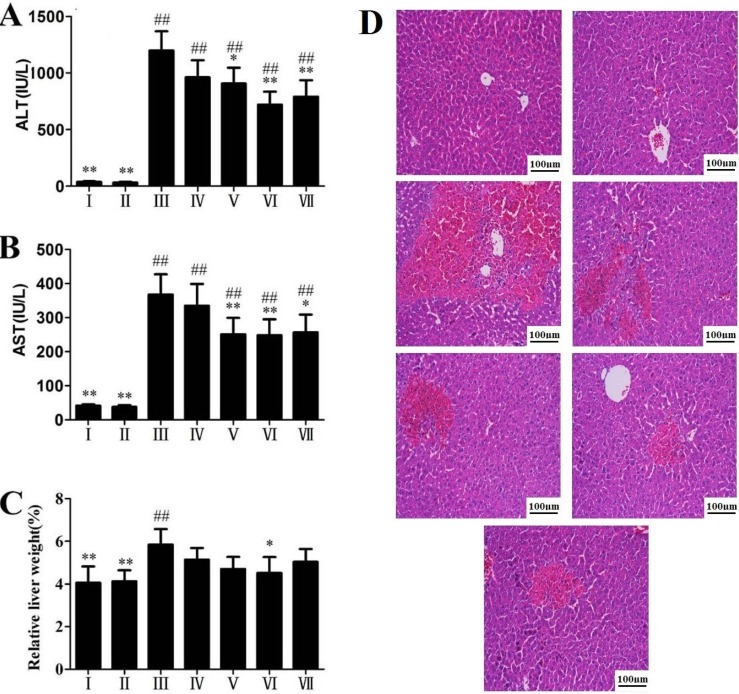
Effects of RLTS on serum ALT and AST activities (**A** and **B**), relative liver weight (**C**) and histopathological examination by hematoxylin and eosin (H&E) staining (**D**) against APAP- induced liver damage in mice. I: RLTS control; II: normal control; III: model; IV: RLTS (100 mg/kg); V: RLTS (200 mg/kg); VI: RLTS (300 mg/kg); VII: silymarin (200 mg/kg). Relative Liver weight (%) = liver weight/body weight × 100. H&E stained sections were observed under 200 × magnification. Values are expressed as mean ± SD in each group. * *p* < 0.05, ** *p* < 0.01 *vs.* model; ^##^ *p* < 0.01 *vs.* normal control.

The activities of ALT and AST in the model group were highly elevated compared with control group (*p* < 0.01), which suggested obvious liver damage caused by APAP (Figure 1A,B). Treatment with RLTS significantly decreased ALT and AST levels in a dose-dependent manner. Relative liver weight increased in the model group (Figure 1C),which was prevented by RLTS at the dose of 300 mg/kg. As shown in Figure 1D, livers from the mice receiving 300 mg/kg of RLTS were morphologically as good as those of the normal mice, and while large hemorrhagic areas, massive confluent necrosis and inflammatory cells infiltration were found in APAP-treated animals, RLTS or silymarin pretreatment attenuated the damage.

### 2.2. Antioxidant Activity of RLTS

**Figure 2:**
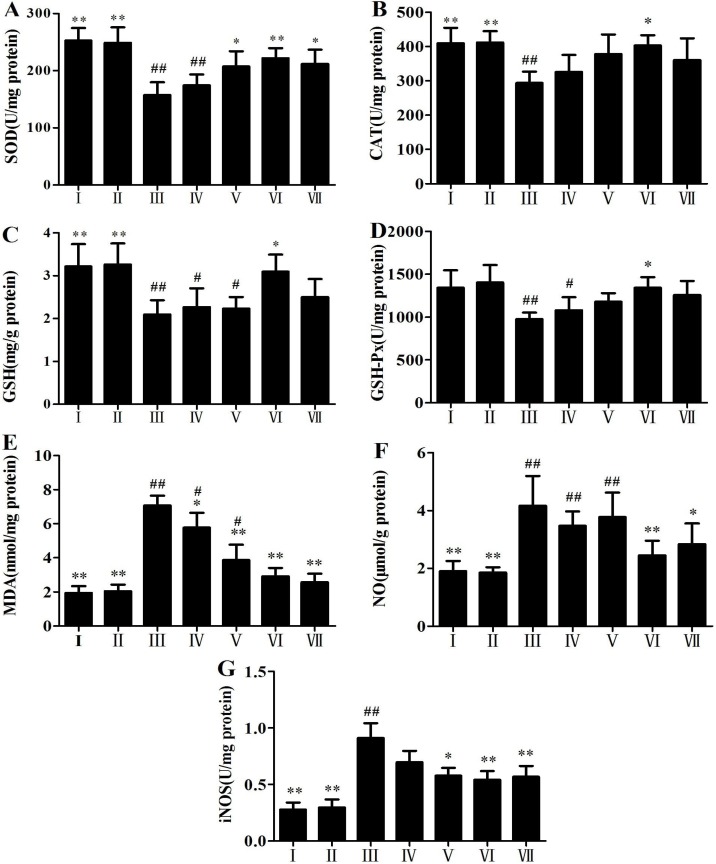
Effects of RLTS on the levels of SOD (**A**), CAT (**B**), GSH (**C**), GSH-Px (**D**), MDA (**E**), NO (**F**) and iNOS (**G**) in livers. I: RLTS control; II: normal control; III: model; IV: RLTS (100 mg/kg); V: RLTS (200 mg/kg); VI: RLTS (300 mg/kg); VII: silymarin (200 mg/kg). Values are expressed as mean ± SD (*n* = 10). * *p* < 0.05, ** *p* < 0.01 *vs.* model group; ^##^ *p* < 0.01, ^#^ *p* < 0.05 *vs.* normal group.

The antioxidant activity results of RLTS are shown in Figure 2A–G. Compared with the control group, the levels of SOD, CAT, GSH and GSH-Px were significantly decreased, and the levels of MDA, NO and iNOS were significantly increased in APAP-treated group, which were all reversed by RLTS (*p* < 0.05).

### 2.3. Effects of RLTS on Nuclei and DNA Fragmentation Caused by APAP

**Figure 3:**
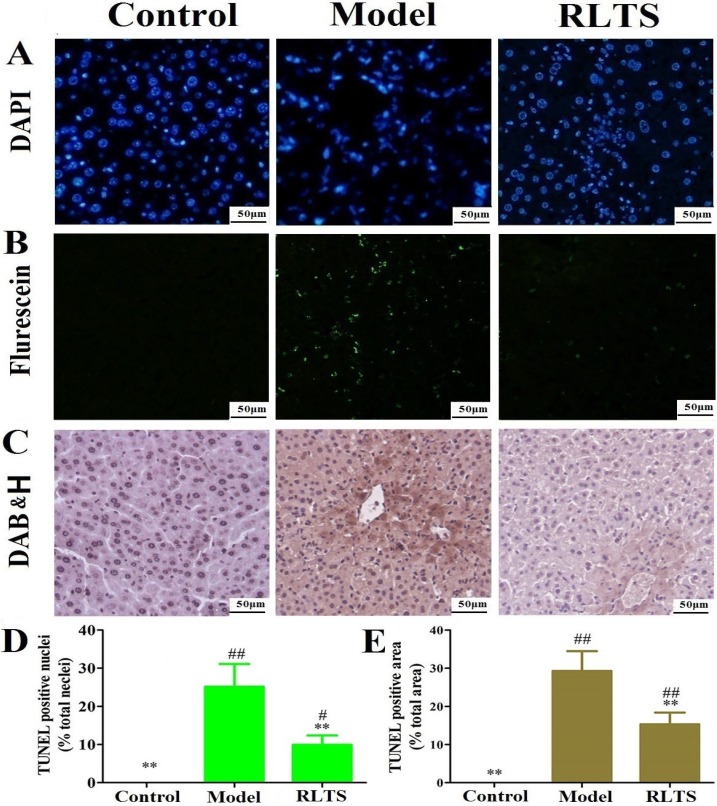
Representative micrographs of DAPI stained nuclei (**A**), TUNEL fluorescent images (**B**) and DAB&H stained images (**C**) in normal control, model and RLTS (300 mg/kg) groups (magnification, 400×). Statistic analysis of TUNEL fluorescent images (**D**) and DAB&H dyeing images (**E**). Values are expressed as mean ± SD (*n* = 4). ** *p* < 0.01 *vs.* model group; ^##^ *p* < 0.01, ^#^ *p* < 0.05 *vs.* normal group.

DAPI staining revealed normal nuclei morphology in the control group as shown in Figure 3A. However in APAP-treated mice condensed nucleus or loss of nuclei was observed. Pretreatment with RLTS assay was used to detect fragmentation of DNA. As shown in Figure 3B,C, TUNEL-positive cells showed green fluorescence and brown after fluorescein labeling and DAB staining respectively. Virtually no TUNEL-positive nuclei was found in control group. Compared with model group, RLTS at the dose of 300 mg/kg significantly reduced the DNA fragmentation (shown in Figure 3D–E).

### 2.4. Effects of RLTS on Cellular Ultrastructure and Mitochondrial Injury

**Figure 4:**
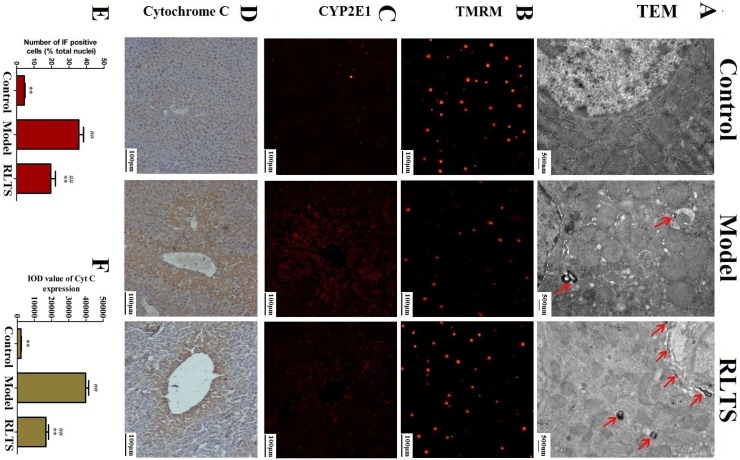
TEM analysis of the livers (magnification, 25,000×) in normal, model and RLTS (300 mg/kg) groups, and the arrows indicated autophagosome (**A**).Mitochondrial membrane potential detected by TMRM staining (**B**). Effects of RLTS (300 mg/kg) on the expression of CYP2E1 (**C**) and cytochrome C (**D**) (magnification, 200×). Statistical analysis of the fluorescence intensity (**E**) and the integrated optical density (**F**). Values are expressed as mean ± SD (*n* = 4). ** *p* < 0.01 *vs.* model group; ^##^ *p* < 0.01 *vs.* normal group.

As shown in Figure 4A the mitochondrial valley was absent and the nucleus was condensed in the model group , which were inhibited by 300 mg/kg of RLTS. Autophagy characterized by autophagosomes was also observed in the model group, which was further enhanced by RLTS. In addition, the mitochondria extracted from the livers were labeled with TMRM. As shown in Figure 4B, RLTS at the dose of 300 mg/kg significantly increased the red fluorescence of TMRM compared with model group indicating decreased impaired mitochondria.

### 2.5. Effect of RLTS on Expressions of CYP2E1 and Cytochrome C

The effects of RLTS on expressions of CYP2E1 and cytochrome C were investigated. As shown in Figure 4C,D APAP administration significantly enhanced the expressions of CYP2E1 and cytochrome C which were all markedly attenuated by the extract. As shown in Figure 4E,F, the declined levels in the RLTS-treated group (300 mg/kg) were 54.29% ± 6.01% and 42.94% ± 7.74% (*p* < 0.01), respectively, compared with the model group.

### 2.6. Effects of RLTS on some Molecular Expressions in Inflammatory Pathway

**Figure 5:**
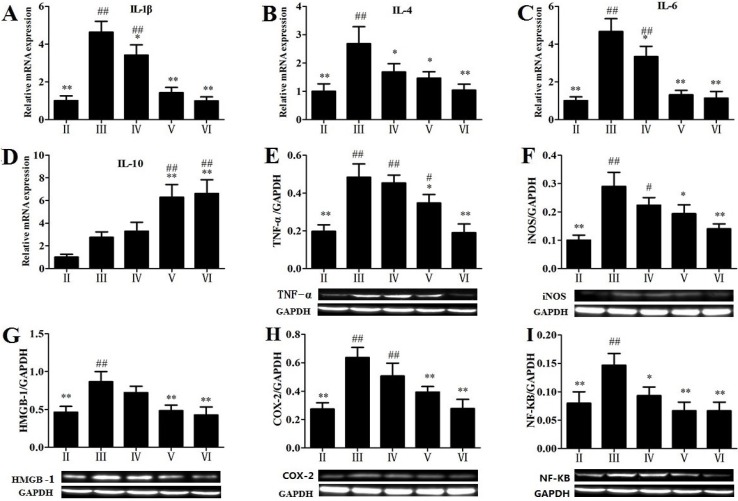
Effects of RLTS on the gene expressions of IL-1β (**A**), IL-4 (**B**), IL-6 (**C**), IL-10 (**D**), and the protein expressions of TNF-α (**E**), iNOS (**F**),HMGB-1 (**G**), COX-2 (**H**), NF-κB (**I**). Values are expressed as mean ± SD (*n* = 4). * *p* < 0.05, ** *p* < 0.01 *vs.* model group; ^##^ *p* < 0.01, ^#^ *p* < 0.05 *vs.* normal group.

As shown in Figure 5, RLTS (300 mg/kg) significantly down-regulated the gene or protein expression of IL-1β, IL-4, IL-6, TNF-α, iNOS, HMGB-1, COX-2 and NF-κB by 4.68-, 2.58-, 4.10-, 2.54-, 2.07-, 2.03-, 2.30- and 1.45-fold. As shown in Figure 5D, the expression of IL-10 was up-regulated by APAP, and however, RLTS pretreatment (200 and 300 mg/kg) markedly further enhanced the gene expression compared with the model group.

### 2.7. Effects of RLTS on Some Molecular Expressions Associated with Apoptosis

**Figure 6:**
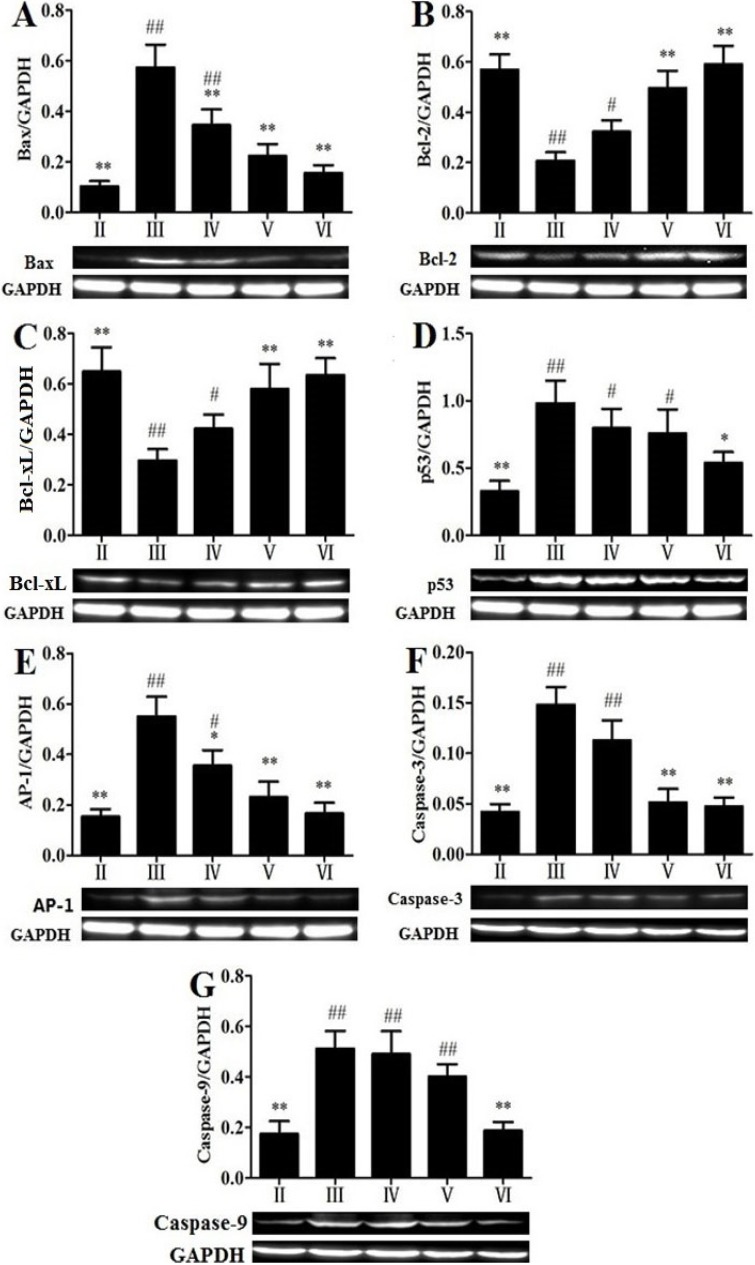
Effects of RLTS on the protein expressions of Bax (**A**), Bcl-2 (**B**), Bcl-xL (**C**), p53 (**D**), AP-1 (**E**), Caspase-3 (**F**), Caspase-9 (**G**). Values are expressed as mean ± SD (*n* = 4). * *p* < 0.05, ** *p* < 0.01 *vs.* model group; ^##^ *p* < 0.01, ^#^ *p* < 0.05 *vs.* normal group.

**Figure 7:**
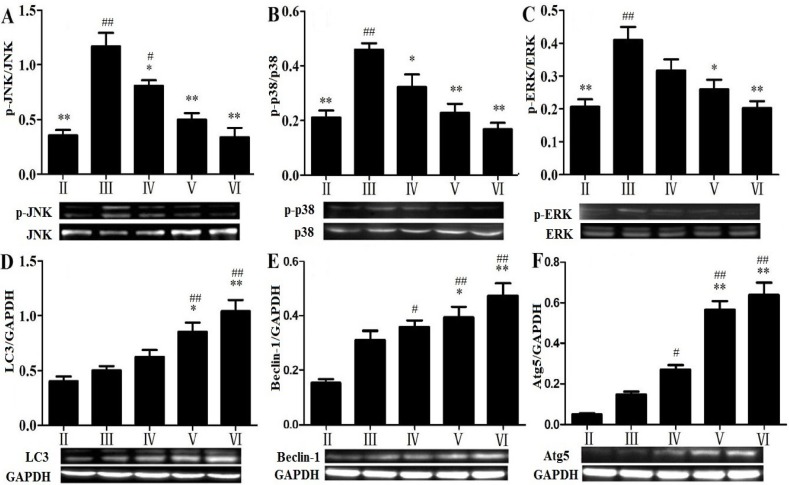
Effects of RLTS on the protein expressions of p-JNK (**A**), p-p38 (**B**), p-ERK (**C**), LC3 (**D**), Beclin-1 (**E**) and Atg5 (**F**). Values are expressed as mean ± SD (*n* = 4). * *p* < 0.05, ** *p* < 0.01 *vs.* model group; ^##^ *p* < 0.01, ^#^ *p* < 0.05 *vs.* normal group.

To investigate the mechanisms of RLTS against APAP-induced liver damage, the apoptotic pathway was examined. As shown in Figure 6A–G , the protein expressions of Bax, p53 and AP-1, as well as Caspase-3 and -9 in APAP-treated group were strongly up-regulated by 3.65-, 2.08-, 3.59-, 2.05-, 2.32-fold, while Bcl-2 and Bcl-xL were down-regulated by 2.85- and 2.19-fold, which were all significantly (*p* < 0.05) reversed by RLTS. In addition, as shown in Figure 7A–C the levels of hepatic p-JNK p-p38 and p-ERK were significantly increased by APAP , which were all significantly down- regulated by RLTS.

### 2.8. Effects of RLTS on Some Molecular Expressions in Autophagic Pathway

As shown in Figure 7D–F, the levels of LC3, Beclin-1 and Atg5 were all increased in the APAP-treated group with no significant differences (*p* > 0.05) compared with the control group. However, RLTS further up-regulated their expressions.

### 2.9. Discussion

APAP overdoses can cause severe liver toxicity [27]. In the present study, RLTS pretreatment significantly lowered the levels of AST and ALT and reduced inflammatory cell infiltration, which meant that RLTS showed significant activity against APAP-induced liver injury.

APAP-induced hepatotoxicity is a complex biological process [28,29]. In the present paper, RLTS significantly improved the levels of GSH, SOD, CAT and GSH-Px, and decreased MDA level, which indicated that the protective effect of RLTS against APAP-induced liver injury may be associated with its antioxidant capacity. NO, a highly reactive oxidant and generated by iNOS, has been considered to be one of the crucial mediator in APAP-induced liver injury [11]. In our work, RLTS inhibited the levels of NO and iNOS, which should be related with the protective effect of RLTS against APAP-induced liver damage.

It has been demonstrated that APAP-induced hepatotoxicity can be modulated by chemicals through affecting P450 activity [30]. CYP2E1 is the primary enzyme for APAP bio-activation and its activity is increased following APAP treatment [2]. In the present work, the expression of CYP2E1 was suppressed by RLTS, which suggested that the protective effect of RLTS against APAP-induced liver injury may be through affecting APAP bio-activation.

Previous study has demonstrated that inflammatory reactions are associated with APAP-induced liver injury [31]. Some pro-inflammatory mediators including TNF-α, IL-1β and IL-6 are potentially harmful because of their uncontrolled and prolonged actions [32], which can be modulated by the activations of NF-κB and HMGB-1 [33,34]. In addition, COX-2, another pro-inflammatory mediator, can produce eicosanoids and cause cellular inflammation and necrosis [35]. Our study revealed that administration of RLTS markedly reduced the expressions of TNF-α, IL-1β, IL-4, IL-6, iNOS, HMGB-1, COX-2 and NF-κB. Furthermore, IL-10 with anti-inflammatory and immunomodulatory effects can attenuate the activation of TNF-α-induced NF-κB pathway [36,37]. Our results showed that APAP increased the expression of IL-10 in the model group, and RLTS further increased its expression. These results indicated that the effects of RLTS against APAP-induced liver injury may be through suppression of inflammation.

Although the pattern of APAP-induced cell death has been generally believed, some evidences have demonstrated that apoptosis may play a major role in the process [38,39]. In the present paper, TUNEL assay and the expressions of some related proteins associated with the effects of RLTS against apoptosis induced by APAP were studied. Cytochrome C is one pro-apoptotic protein [28], and the leakage of it into cytosol in APAP-treated mice has been found [9]. The release of cytochrome C means the apoptosis [40]. Moreover, AP-1, a crucial transcription factor, can promote hepatocyte apoptosis through cytochrome C release and caspase-3 activation [33]. Bcl-2 and Bcl-xL can be as the antioxidants and exert anti-apoptotic activities [38], whereas Bax can exhibit conformational change under apoptotic process [41]. p53 can reduce the expression of Bcl-2 and up-regulate the expression of Bax to complete the promotion of apoptosis [42]. In the present paper, the leakage of cytochrome C and the expressions of caspase-3, -9 and AP-1 were attenuated by RLTS, and the extract up-regulated the expressions of Bcl-2, Bcl-xL, and down-regulated the expressions of Bax, p53. These findings suggested that the action of the extract against APAP-induced liver injury may be through protection of apoptosis.

The MAP kinase family plays important roles in regulation of cell proliferation and cell death [43]. It has been confirmed that oxidative stress derived from APAP bioactivation can directly activate JNK pathway, and phosphorylated JNK can block the anti-apoptotic function of Bcl-2 family [5]. Similarly, phosphorylations of p38 and ERK are responsible for shifting the balance of the proapoptotic and antiapoptotic members of Bcl-2 family proteins, which may alter mitochondrial membrane permeabilization and finally cause cytochrome C release [44]. In the study, RLTS down-regulated the phosphorylation levels of JNK, p38 and ERK, which indicated that the action of the extract against APAP-induced liver injury may be through affecting MAPK signal pathway.

Apart from apoptotic and necrotic cell death, autophagy is an evolutionarily conserved pathway that involves the sequestration and delivery of cytoplasmic material to lysosome [45], which can play protective roles in some diseases [46]. LC3, one microtubule-associated protein, has been used as one marker for the mammalian autophagosome [46]. In the elongation/enclosure step of autophagosome formation, Atg5 and Atg16 can form the Atg12-Atg5-Atg16 complex [47]. Furthermore, Beclin-1 interacted with the anti-apoptotic members of Bcl-2 and Bcl-xL can activate autophagy [48]. In the present study, the increased levels of Beclin-1, Atg5 and LC3 induced by APAP were all further up-regulated by RLTS, which suggested that the autophagy activated by RLTS may be beneficial to prevent APAP-induced liver damage.

## 3. Experimental

### 3.1. Plant Material and Preparation of RLTS

*Rose laevigata* Michx fruit was obtained from Yun-nan Qiancaoyuan Pharmaceutical Company Co. Ltd. (Kunming, China) and authenticated by Dr. Yunpeng Diao (College of Pharmacy, Dalian Medical University, Dalian, China). A voucher specimen (DLMU, JYZ-2012080426) was deposited in the Herbarium of College of Pharmacy, Dalian Medical University (Dalian, China). Total saponins from *R. laevigata* Michx fruit (RLTS) was prepared and the content of saponin in the crude extract was determined [49].

### 3.2. Chemical and Reagents

APAP with the purity of >98% was obtained from HEOWNS (Tianjin, China). Silymarin was purchased from Sigma Chemical Company (Milan, Italy). Detection kits including AST, ALT, CAT, SOD, GSH, GSH-Px, MDA, NO and iNOS were all supplied by Nanjing Jiancheng Institute of Biotechnology (Nanjing, China). Tissue Protein Extraction Kit was produced by KeyGEN Biotech. Co., Ltd. (Nanjing, China). Enhanced Bicinchoninic Acid (BCA) Protein Assay Kit was purchased from Beyotime Institute of Biotechnology (Haimen, China). Tris, SDS and 4',6'-Diamidino-2-phenylindole (DAPI) were obtained from Sigma (St. Louis, MO, USA). Hematoxylin, Histostain-TM-Plus Kit and 3',3'-diaminobenzidine tetrahydrochloride (DAB) Substrate Kit were provided by Zhongshan Golden Bridge Biotechnology (Beijing, China). In Situ Cell Death Detection Kit, POD was supplied by Roche Diagnostics (Roche Diagnostics Gmbh, Mannheim, Germany). Tetramethylrhodamine methyl ester (TMRM) was purchased from Life Technologies (Eugene, OR, USA). RNAiso Plus, PrimeScript^®^ RT reagent Kit with gDNA Eraser (Perfect Real Time) and SYBR^®^ *Premix Ex Taq*™ II (Tli RNaseH Plus) were purchased from TaKaRa Biotechnology Co., Ltd. (Dalian, China). Antibody against CYP2E1 was provided by US Biologicals (Swampscott, MA, USA). The antibodies against p38, p-p38, ERK, p-ERK, JNK and p-JNK were supplied by Bioworld Technology (Louis Park, MN, USA). Other primary antibodies and horseradish peroxidase-conjugated goat anti-rabbit IgG, and horseradish peroxidase-conjugated goat anti-mouse IgG antibodies were all provided by Proteintech Group (Chicago, IL, USA) and Boster Biological Technology (Wuhan, China). TRITC-conjugated goat anti-rabbit IgG was purchased from Zhongshan Golden Bridge Biotechnology (Beijing, China).

### 3.3. Animals

Male Kunming mice (18–22 g) were provided by the Experimental Animal Center of Dalian Medical University, Dalian, China (Quality certificate number: SCXK (Liao) 2008–0002). The mice were housed in a climate-controlled room with relative humidity (60% ± 5%), temperature (21 ± 3 °C), 12 h light-dark cycle. They were fed with commercial pelleted feed from Xietong Organism Institute (Nanjing, China) and water *ad libitum*. The animals were acclimatized at least one week prior to experiments and treated in accordance with the guidelines recommended by China National Institutes of Healthy Guidelines for the Care and Use of Laboratory Animals.

### 3.4. Experimental Design

The animals were randomly divided into seven groups (*n* = 10). Group I (RLTS control, 300 mg/kg), Group II (normal control group), Group III (model group), Groups IV-VI (RLTS-treated groups) and Group VII (positive control group). Groups IV, V and VI were administrated RLTS at the doses of 100, 200 and 300 mg/kg suspended in 0.5% carboxymethylcellulose sodium (CMC-Na) and Group VII was given 200 mg/kg of silymarin once daily for 7 consecutive days, respectively. Two hour after final administration, the mice in Groups III-VII were injected intraperitoneally with acetaminophen (400 mg/kg), while the mice in Groups I and II received only appropriate vehicle. The animals were anesthetized for collecting the blood and sacrificed to obtain the livers after 24 h acetaminophen challenge followed by fasting. The fresh liver was weighed to count the relative liver weight (relative liver weight (%) = liver weight/body weight × 100). A portion of liver was cut and fixed in 10% formalin and the remaining parts were immediately stored at −80 °C.

### 3.5. Serum Biochemistry

The activities of ALT and AST in serum were evaluated by detection kits according to the manufacturer’s instructions.

### 3.6. Histopathological Examination

After fixing in 10% neutral formalin solution, the tissues were dehydrated with a sequence of ethanol solutions, embedded in paraffin wax and cut at 5 μm thickness, stained with haematoxylin and eosin (H&E stain) and then observed for morphological evaluation under a light microscope (Leica DM4000B, Solms, Germany).

### 3.7. Determinating the Levels of Antioxidant Markers in Liver Tissue

The liver tissues were homogenized in cold Tris-HCl to make 1:9 (w/v) homogenates. The homogenate was centrifuged (2500 *g* for 10 min at 4 °C) and the supernatants were collected. The levels of SOD, CAT, GSH, GSH-Px, NO and iNOS were detected according to the instructions of the kits.

### 3.8. Assay for Lipid Peroxidation Product

The lipid peroxidation was estimated by MDA level, which was determined under the manufacturer’s instruction.

### 3.9. DAPI Staining

Morphological assessment of nuclei was performed with DAPI staining as described previously [50]. Briefly, the paraffin sections were deparaffinized with xylene and rehydrated with different concentrations of alcohol. Then, the slices were incubated with 1 μg/mL DAPI for 8 min, washed with PBS and examined by fluorescence microscopy (Olympus BX63, Tokyo, Japan).

### 3.10. TUNEL Assay

TUNEL assay was carried out with paraffin-embedded slices from control, model and RLTS-treated (300 mg/kg) groups using a commercial kit according to the manufacturer’s instructions. Briefly, the dewaxed course was treated as described above. After incubation with Proteinase K for 8 min, the green fluorescein labeled dUTP solution was added on the surface of sections and incubated at 37 °C for 1 h. Then the slides were washed and photographed using fluorescence microscopy. Total of 50 μL converter-POD was added on the tissues for the reaction at 37 °C for 30 min. Then the samples were spotted with DAB fluid and hematoxylin. Images were obtained by using fluorescence microscopy (Olympus BX63) and inverted digital imaging light microscopy (Leica DM4000B). TUNEL positive areas showed green fluorescence or brown staining, and the damage degree of DNA was identified by the mean number of positive cells.

### 3.11. Transmission Electron Microscopy (TEM) Assay

Fresh liver tissues from control, model and RLTS (300 mg/kg) groups were perfused with 2% glutaraldehyde overnight at 4 °C. The samples were treated as the previous description [28], and the ultramicrotomies were observed and imaged by using an electron microscope (JEM-2000EX, JEOL, Sagamihara, Japan).

### 3.12. Determination of Mitochondria Membrane Potential (ΔΨm)

Mitochondria were isolated from liver tissue according to the manufacturer’s instruction. Briefly, total of 100 mg liver tissue was gained from the mice in control, model and RLTS (300 mg/kg) groups. Then the tissue was made into 10% homogenate with separating reagent A and centrifuged at 600 *g* for 5 min at 4 °C to generate the supernatants. After that, the supernatants were centrifuged at 11,000 *g* for 10 min at 4 °C, and the sediment (mitochondria) was obtained. After incubation with tetramethylrhodamine methylester (TMRM) in dark for 30 min at 37 °C, the mitochondrial membrane potential was detected by the red fluorescence and photographed by using a fluorescence microscope (Olympus BX63).

### 3.13. Immunofluorescence and Immunohistochemical Assays for CYP2E1 and Cytochrome C

The liver sections were deparaffinized and rehydrated as described above, and then treated with 0.01 mol/L citrate (pH = 6.0) in a microwave oven for 15 min. Thereafter, 3% hydrogen peroxide (H_2_O_2_) was used to block endogenous peroxidase activity for 10 min and normal goat serum was used to block nonspecific protein binding for 20 min. Then, the slices were incubated at 4 °C overnight with the rabbit anti-CYP2E1 antibody (1:100, dilution) or rabbit anti-cytochrome C antibody (1:100 dilution), followed by incubation with TRITC-conjugated goat anti-rabbit IgG for 30 min, or biotinylated goat anti-rabbit IgG and horseradish peroxidase-conjugated streptavidin for 15 min. The images were obtained by a light microscope (Nikon Eclipse TE2000-U, NIKON, Tokyo, Japan).

### 3.14. Quantitative Real-time PCR

Total RNA was isolated from liver tissues with RNAiso Plus reagent, then total RNA was reverse-transcribed into cDNA by using PrimeScript^®^ RT reagent Kit with a TC-512 PCR system (TECH-NE, Stone, UK). Thereafter, the aliquots of cDNAs were amplified using specific primers. The sequences of the primers were as follows: GAPDH: forward, 5'-TGTGTCCGTCGTGGATCTGA-3', reverse, 5'-TTGCTGTTGAAGTCGCAGGAG-3', (NM_008084.2); IL-1β: forward, 5'-TCCAGGAT GAGGACATGAGCAC-3', reverse, 5'-GAACGTCACACACCAGCAGGTTA-3', (NM_008361.3); IL-4: forward, 5'-ACGGAGATGGATGTGCCAAAC-3', reverse, 5'-AGCACCTTGGAAGCCCTA CAGA-3', (NM_021283.2); IL-6: forward, 5'-CCACTTCACAAGTCGGAGGCTTA-3', reverse, 5'-CCAGTTTGGTAGCATCCATCATTTC-3', (NM_031168.1); IL-10: forward, 5'-GCCAGAGCC ACATGCTCC -TA-3', reverse, 5'-GATAAGGCTTGGCAACCCAAGTAA-3', (NM_010548.2). The levels of mRNA expressions were assessed by real-time PCR with SYBR^®^ Premix Ex Taq™ II (Tli RNaseH Plus) and 7500 Real Time PCR System (Applied Biosystems, Carlsbad, CA, USA). A no-template control was analyzed in parallel for each gene, and GAPDH gene was amplified as a housekeeping gene. At last, the unknown template was calculated through the standard curve for quantitative analysis.

### 3.15. Western Blotting Assay

Total protein was isolated using the tissue protein extraction kit based on the manufacturer’s instruction and the content of protein was determined. An aliquot of the supernatant was separated by electrophoresis with SDS-PAGE gel and transferred to a PVDF membrane (Millipore, Billerica, MA, USA). After blocking in 5% dried skim milk for 3 h, the membrane was individually incubated overnight at 4 °C with primary antibodies including rabbit anti-TNF-α (1:400), rabbit anti-iNOS (1:500), rabbit anti-HMGB-1 (1:500), rabbit anti-COX-2 (1:1000), anti-NF-κB (1:700), rabbit anti-Bax (1:700), rabbit anti-Bcl-2 (1:700), rabbit anti-Bcl-xL (1:500), rabbit anti-p53 (1:500), rabbit anti-AP-1 (1:1000), rabbit anti-Caspase-3 (1:200), rabbit anti-Caspase-9 (1:200), rabbit anti-p-JNK (1:500), rabbit anti-JNK (1:500), rabbit anti-p-p38 (1:500), rabbit anti-p38 (1:500), rabbit anti-p-ERK (1:500), rabbit anti-ERK (1:500), rabbit anti-LC3 (1:1000), rabbit anti-beclin-1 (1:1000), rabbit anti-Atg5 (1:500), mouse anti- GAPDH (1:1000). Then the blots were incubated with secondary antibodies either the goat anti-rabbit (1:1000 dilution) or the goat anti-mouse (1:2000 dilution) IgG-horseradish peroxidase-conjugated for 3 h at room temperature. Detection was performed by using an enhanced chemiluminescence (ECL) method and photographed by Bio-Spectrum Gel Imaging System (UVP, Upland, CA, USA). To remove the variations due to protein quantity and quality, the data were revised to GAPDH expression (IOD of objective protein *versus* IOD of GAPDH protein).

### 3.16. Statistical Analysis

Data were analyzed by using the SPSS software 17.0. Differences between the groups were performed by one-way ANOVA and Tukey *post hoc* tests, using *p* < 0.05 and *p* < 0.01 as the level of significance. Values were expressed as mean ± standard deviation (SD).

## 4. Conclusions

In summary, the present study suggests that total saponins from *Rosa laevigata* Michx fruit shows a significant protective effect against APAP-induced liver damage in mice via induction of autophagy, and suppression of inflammation and apoptosis. However, further studies should be carried out to investigate in depth the mechanisms, drug targets and the active compounds of the crude extract useful for treatment of liver injury.

## Abbreviations

RLTS: *Rosa laevigata* Michx fruit
APAP: acetaminophen
ALT: alanine transaminase
AST: aspartate transaminase
MDA: malondialdehyde
NO: nitric oxide
iNOS: inducible nitric oxide synthase
SOD: superoxide dismutase
CAT: catalase
GSH: glutathione
GSH-Px: glutathione peroxidase
TUNEL: TdT-mediated dUTP nick end labeling
TEM: transmission electron microscopy
IL-1β: interleukin 1β
IL-4: interleukin
IL-6: interleukin 6
TNF-α: tumor necrosis factor α
Bax: Bcl-2 Assaciated X protein
HMGB-1: High-mobility group 1
COX-2: cyclooxygenase-2
NF-κB: nuclear factor kappa B
AP-1: activator protein-1
IL-10: interleukin 10
Bcl-2: B-cell lymphoma-2
Bcl-xL: B-cell lymphoma-extra large
LC3: light chain 3
Atg5: autophagy protein 5

## References

[1] Abraham P. (2005). Oxidative stress in paracetamol-induced pathogenesis: (I)-Renal Damage. Indian J. Biochem. Biophys..

[2] Slitt A.M., Dominick P.K., Roberts J.C., Cohen S.D. (2005). Effect of ribose cysteine pretreatment on hepatic and renal acetaminophen metabolite formation and glutathione depletion. Basic Clin. Pharmacol. Toxicol..

[3] Jaeschke H., Knight T.R., Bajt M.L. (2003). The role of oxidant stress and reactive nitrogen species in acetaminophen hepatotoxicity. Toxicol. Lett..

[4] James L.P., Mayeux P.R., Hinson J.A. (2003). Acetaminophen-induced hepatotoxicity. Drug Metab. Dispos..

[5] Latchoumycandane C., Goh C.W., Ong M.M., Boelsterli U.A. (2007). Mitochondrial Protection by the JNK Inhibitor Leflunomide Rescues Mice from Acetaminophen-induced Liver Injury. Hepatology.

[6] Burcham P.C., Harman A.W. (1991). Acetaminophen toxicity results in site-specific mitochondrial damage in isolated mouse hepatocytes. J. Biol. Chem..

[7] Harman A.W., Kyle M.E., Serroni A., Farber J.L. (1991). The killing of cultured hepatocytes by *N*-acetyl-p-benzoquinone imine (NAPQI) as a model of the cytotoxicity of acetaminophen. Biochem. Pharmacol..

[8] Jaeschke H., Farhood A., Smith C.W. (1990). Neutrophils contribute to ischemia/reperfusion injury in rat liver *in vivo*. FASEB J..

[9] Masubuchi Y., Suda C., Horie T. (2005). Involvement of mitochondrial permeability transition in acetaminophen-induced liver injury in mice. J. Hepatol..

[10] Adamson G.M., Harman A.W. (1993). Oxidative stress in cultured hepatocytes exposed to acetaminophen. Biochem. Pharmacol..

[11] Gardner C.R., Heck D.E., Yang C.S., Thomas P.E., Zhang X.J., DeGeorge G.L., Laskin J.D., Laskin D.L. (1998). Role of nitric oxide in acetaminophen-induced hepatotoxicity in the rat. Hepatology.

[12] Lemasters J.J., Nieminen A.L., Qian T., Trost L.C., Elmore S.P., Nishimura Y., Crowe R.A., Cascio W.E., Bradham C.A., Brenner D.A. (1998). The mitochondrial permeability transition in cell death: A common mechanism in necrosis, apoptosis and autophagy. Biochim. Biophys. Acta.

[13] Blazka M.E., Elwell M.R., Holladay S.D., Wilson R.E., Luster M.I. (1996). Histopathology of acetaminophen-induced liver changes: Role of interleukin 1 alpha and tumor necrosis factor alpha. Toxicol. Pathol..

[14] Levine B. (2005). Eating oneself and uninvited guests: Autophagy-related pathways in cellular defense. Cell.

[15] Sabiba E.P., Rasool M., Vedi M., Navaneethan D., Ravichander M., Parthasarathy P., Thella S.R. (2013). Hepatoprotective and antioxidant potential of Withania somnifera against paracetamol-induced liver damage in rats. Int. J. Pharm. Pharm. Sci..

[16] Bajt M.L., Knight T.R., Lemasters J.J., Jaeschke H. (2004). Acetaminophen-induced oxidant stress and cell injury in cultured mouse hepatocytes: Protection by N-acetyl cysteine. Toxicol. Sci..

[17] Smilkstein M.J., Knapp G.L., Kulig K.W., Rumack B.H. (1988). Efficacy of oral N– acetylcysteine in the treatment of acetaminophen overdose. Analysis of the national multicenter study (1976 to 1985). N. Engl. J. Med..

[18] Zhao X., Cong X., Zheng L., Xu L., Yin L., Peng J. (2012). Dioscin, a natural steroid saponin, shows remarkable protective effect against acetaminophen-induced liver damage* in vitro* and *in vivo*. Toxicol. Lett..

[19] Muriel P., Garciapina T., Perez-Alvarez V., Mourelle M. (1992). Silymarin protects against paracetamol-induced lipid peroxidation and liver damage. J. Appl. Toxicol..

[20] Kim Y.W., Ki S.H., Lee J.R., Lee S.J., Kim C.W., Kim S.C., Kim S.G. (2006). Liquiritigen -in, an aglycone of liquiritin in Glycyrrhizae radix, prevents acute liver injuries in rats induced by acetaminophen with or without buthionine sulfoximine. Chem. Biol. Interact..

[21] Ghosh J., Das J., Manna P., Sil P.C. (2010). Arjunolic acid, a triterpenoid saponin, prevents acetaminophen (APAP)-induced liver and hepatocyte injury via the inhibition of APAP bioactivation and JNK-mediated mitochondrial protection. Free Radic. Biol. Med..

[22] Zhang T.Y., Nie L.W., Wu B.J., Yang Y., Zhao S.S., Jin T. (2004). Hypolipedemic activity of the polysaccharose from *Rosa laevigata* Michx fruit. Chin. J. Public. Health.

[23] Zou H.T., Yang Y., Chen S.J. (2006). The resource values and prospective of* Rosa laevigata* Michx fruits as beverage. Technol. Food Ind..

[24] Zhao Y.T., Guo X.M., Li F.Z. (2003). Antioxidative activity of polysaccharide from *Rosa laevigata* Michx. J. Biol..

[25] Francis G., Kerem Z., Makkar H.P., Becke K. (2002). The biological action of saponins in animal systems: A review. Br. J. Nutr..

[26] Dong D.S., Zhang S., Yin L.H., Tang X.Q., Xu Y.W., Han X., Qi Y., Peng J.Y. (2013). Protective effects of the total saponins from *Rosa laevigata* Michx fruit against carbon tetrachloride-induced acute liver injury in mice. Food Chem. Toxicol..

[27] Thomas S.H. (1993). Paracetamol (acetaminophen) poisoning. Pharmacol. Ther..

[28] Kass G.E. (2006). Mitochondrial involvement in drug-induced hepatic injury. Chem. Biol. Interact..

[29] Hinson J.A., Roberts D.W., James L.P. (2010). Mechanisms of Acetaminophen-Induced Liver Necrosis. Handb. Exp. Pharmacol..

[30] Raucy J.L., Lasker J.M., Lieber C.S., Black M. (1989). Acetaminophen activation by human liver cytochromes P450IIE1 and P450IA2. Arch. Biochem. Biophys..

[31] Ishida Y., Kondo T., Tsuneyama K., Lu P., Takayasu T., Mukaida N. (2004). The pathogenic roles of tumor necrosis factor receptor p55 in acetaminophen-induced liver injury in mice. J. Leukoc. Biol..

[32] Aldaba-Muruato L.R., Moreno M.G., Shibayama S., Tsutsumi V., Muriel P. (2012). Protective effects of allopurinol against acute liver damage and cirrhosis induced by carbon tetrachloride: Modulation of NF-κB, cytokine production and oxidative stress. Biochim. Biophys. Acta.

[33] Taki-Eldin A., Zhou L., Xie H.Y., Chen K.J., Yong He D.Y., Zheng S.S. (2012). Triiodothyronine attenuates hepatic ischemia/reperfusion injury in a partial hepatectomy model through inhibition of proinflammatory cytokines, transcription factors, and adhesion molecules. J. Surg. Res..

[34] Watanabe T., Kubota S., Nagaya M., Ozaki S., Nagafuchi H., Akashi K., Taira Y., Tsukikawa S., Oowada S., Nakano S. (2005). The Role of HMGB-1 on the Development of Necrosis During Hepatic Ischemia and Hepatic Ischemia/Reperfusion Injury in Mice. J. Surg. Res..

[35] Hu K.Q. (2003). Cyclooxygenase 2 (COX2)-prostanoid pathway and liver diseases. Prostaglandins Leukot. Essent. Fatty Acids.

[36] Bourdi M., Masubuchi Y., Reilly T.P., Amouzadeh H.R., Martin J.L., George J.W., Shah A.G., Pohl L.R. (2002). Protection against acetaminophen-induced liver injury and lethality by interleukin 10: Role of inducible nitric oxide synthase. Hepatology.

[37] Dhingra S., Sharma A.K., Arora R.C., Slezak J., Singal P.K. (2009). IL-10 attenuates TNF-alpha-induced NF kappaB pathway activation and cardiomyocyte apoptosis. Cardiovasc. Res..

[38] Ray S.D., Mumaw V.R., Raje R.R., Fariss M.W. (1996). Protection of acetaminophen-induced hepatocellular apoptosis and necrosis by cholesteryl hemisuccinate pretreatment. J. Pharmacol. Exp. Ther..

[39] Ferret P.J., Hammoud R., Tulliez M., Tran A., Trebeden H., Jaffray P., Malassagne B., Calmus Y., Weill B., Batteux F. (2001). Detoxification of reactive oxygen species by a nonpeptidyl mimic of superoxide dismutase cures acetaminophen-induced acute liver failure in the mouse. Hepatology.

[40] Cai J., Yang J., Jones D.P. (1998). Mitochondrial control of apoptosis: The role of cytochrome c. Biochim. Biophys. Acta.

[41] Ding W.X., Nam O.C. (2003). Role of oxidative stress and mitochondrial changes in cyanobacterial- induced apoptosis and hepatotoxicity. FEMS Microbiol. Lett..

[42] Weng S.Y., Yang C.Y., Li C.C., Sun T.P., Tung S.Y., Yen J.Y. (2011). Synergism between p53 and Mcl-1 in protecting from hepatic injury, fibrosis and cancer. J. Hepatol..

[43] Kim H.Y., Park J., Lee K.H., Lee D.U., Kwak J.H., Kim Y.S., Lee S.M. (2011). Ferulic acid protects against carbon tetrachloride-induced liver injury in mice. Toxicology.

[44] Bhattacharyya S., Pal P.B., Sil P.C. (2013). A 35 kD Phyllanthus niruri protein modulates iron mediated oxidative impairment to hepatocytes via the inhibition of ERKs, p38 MAPKs and activation of PI3k/Akt pathway. Food Chem. Toxicol..

[45] Pattingre S., Tassa A., Qu X., Garuti R., Liang X.H., Mizushima N., Packer M., Schneider M.D., Levine B. (2005). Bcl-2 Antiapoptotic Proteins Inhibit Beclin 1-Dependent Autophagy. Cell.

[46] Yorimitsu T., Klionsky D.J. (2005). Autophagy: Molecular machinery for self-eating. Cell Death Differ..

[47] Ohsumi Y. (2001). Molecular dissection of autophagy: Two ubiquitin-like systems. Nat. Rev. Mol. Cell Biol..

[48] Lavallard V.J., Meijer A.J., Codogno P., Gual P. (2012). Autophagy, signaling and obesity. Pharmacol. Res..

[49] Zhang S., Lu B.N., Han X., Xu L.N., Qi Y., Yin L.H., Xu Y.W., Zhao Y.Y., Liu K.X., Peng J.Y. (2013). Protection of the flavonoid fraction from *Rosa laevigata* Michx fruit against carbon tetrachloride-induced acute liver injury in mice. Food Chem. Toxicol..

[50] Jia Y.N., Ji L., Zhang S., Xu L.N., Yin L.H., Li L., Zhao Y.Y., Peng J.Y. (2012). Total flavonoids from *Rosa Laevigata* Michx fruit attenuates hydrogen peroxide induced injury in human umbilical vein endothelial cells. Food Chem. Toxicol..

